# Dyspnea Management in Patients Presenting to the Emergency Department at Cantonal Hospital Baselland—A Retrospective Observational Study and Medical Audit

**DOI:** 10.3390/jcm14041378

**Published:** 2025-02-19

**Authors:** Emanuele Debernardi, Fabienne Jaun, Maria Boesing, Joerg Daniel Leuppi, Giorgia Lüthi-Corridori

**Affiliations:** 1 University Institute of Internal Medicine, Cantonal Hospital Baselland, CH 4410 Liestal, Switzerland; emanuele.debernardi@ksbl.ch (E.D.); fabienne.jaun@ksbl.ch (F.J.); giorgia.luethi-corridori@ksbl.ch (G.L.-C.); 2 Faculty of Medicine, University of Basel, CH 4056 Basel, Switzerland

**Keywords:** dyspnea, audit, emergency department, clinical assessment

## Abstract

**Background/Objectives:** Dyspnea, the subjective experience of breathing discomfort, accounts for approximately 5% of emergency department (ED) presentations, 10% of general ward admissions, and 20% of intensive care unit (ICU) admissions. Despite its prevalence, dyspnea remains a challenging clinical manifestation for physicians. To the best of our knowledge, there are no international guidelines for the assessment and management of patients with dyspnea coming to the ED. In this study, we aim to evaluate how dyspnea cases are assessed and managed at Cantonal Hospital Baselland in Liestal (KSBL) and to audit these practices. **Methods:** We conducted a retrospective, observational study of hospital records from KSBL, including all patients presenting to the ED with dyspnea as their primary symptom who were subsequently admitted to the internal medicine ward for at least one night between January and December 2022. Data on assessment and management practices were compared using the medStandards algorithm. **Results:** A total of 823 cases were included. The median age at admission was 76 years (with a range of 15–99), and 57% of the patients were male. Blood pressure and heart rate were documented in 93.8% of the cases, respiratory rate in 61.4%, oxygen saturation in 96.1%, and body temperature in 86.3%. The patient’s subjective dyspnea description was recorded in 14.8% of the cases, while the temporal onset (timing of symptoms) was documented in 98.8%, and the intensity of effort triggering dyspnea was noted in 36.2% of cases. A dyspnea index scale was used in 7.8% and smoking status was documented in 41.1% of the cases. Lung percussion was performed in 2.6% of the cases, while a lung auscultation was performed in 94.4% and a heart auscultation was performed in 85.3% of cases. A complete blood count with a basic metabolic panel and TSH test was collected in 86.9% of the cases, while a blood gas analysis was collected in 34.0% of the cases. An ECG was reported in 87.5% of the cases. From the 337 patients who should have received an emergency ultrasound, 10.1% received one. The three most frequent final diagnoses were decompensated heart failure (28.4%), pneumonia (26.4%), and COVID-19 (17.0%). None of the three patients with a known neuromuscular disease were admitted to the shock room. **Conclusions:** Our findings reveal that the medStandards algorithm was only partially followed at the ED in KSBL Liestal, highlighting gaps in detailed history taking, respiratory rate measurement, lung percussion, and emergency ultrasound use. Given the frequency of dyspnea-related presentations, systematic improvements in the adherence to assessment protocols are urgently needed to enhance patient outcomes.

## 1. Introduction

Dyspnea refers to various uncomfortable sensations related to breathing, including a sense of air hunger, a perception of effort, and a feeling of tightness in the chest [1,2]. According to the American Thoracic Society, dyspnea is described as “a subjective experience of breathing discomfort that consists of qualitatively distinct sensations that vary in intensity” and can differ depending on the underlying pathophysiological mechanism, and its perception is influenced by the patient’s social, cultural, and psychological characteristics [3]. Similar to pain, dyspnea is a symptom rather than a clinical sign, and its accurate assessment relies on self-reporting, as only the individual experiencing it can perceive it [3]. Clinical signs of respiratory distress, such as tachypnea, the use of accessory muscles, and intercostal retractions, must be consequently carefully distinguished from the subjective experience of dyspnea [4,5,6,7,8]. Dyspnea is a debilitating symptom, second only to pain in its impact, affecting up to a 25% of the general population and 50% of severely ill patients [9,10,11,12,13]. It is also associated with decreased functional status and poorer psychological health in older individuals [14], contributing to low adherence to exercise training programs in sedentary adults [15] and patients with chronic obstructive pulmonary disease (COPD) [16]. Dyspnea is considered acute when it develops over hours to days. However, patients with chronic conditions may experience acute exacerbation triggered by a new concomitant illness or a worsening of the original disease [17]. Acute dyspnea is a leading cause of emergency department (ED) presentations [18], occurring in a wide range of conditions, including cardiorespiratory, infectious, and oncological diseases [19]. In the United States alone, nearly six million ED visits in 2021 were attributed to acute dyspnea, highlighting it as a significant public health concern [20]. Dyspnea accounts for approximately 5% of ED presentations, around 10% of admissions to general hospital wards, and 20% of admissions to intensive care units (ICUs) [21]. Epidemiological studies of dyspneic patients have revealed that the primary ED diagnoses include pneumonia (approximately 25%), heart failure (around 18%), COPD exacerbation (approximately 15%), and asthma (about 10%), with an overall in-hospital mortality rate of 5% [22]. In individuals of advanced age, there is a higher incidence of dyspnea of a cardiac origin, COPD exacerbation, and pulmonary embolism (PE), as well as an increase in patients with more than two diagnoses or exacerbations of chronic disease [23,24]. Dyspnea is caused by a broad spectrum of potential diagnoses, requiring a prompt assessment that pays special attention to various elements of the patient’s medical history, physical examination, blood biomarkers, and radiological evaluations [17]. Its intensity upon presentation at the ED is a predictor of hospital admission [25], and it is associated with a long length of stay in the ED [26], along with a high in-hospital fatality rate [26,27]. Emerging evidence suggests that in certain cases, dyspnea is more closely correlated with 5-year mortality than forced expiratory volume in 1 s (FEV1) in individuals with chronic lung disease [28]. Dyspnea is also more closely associated with cardiac mortality than angina pectoris [29].

Despite its prevalence, dyspnea remains a complex clinical challenge for physicians, particularly in the ED setting. For these reasons, it is essential to audit the management of patients at the ED. To the best of our knowledge, currently, there are no specific international guidelines for the assessment and management of patients with dyspnea coming to the ED. This study has two primary objectives: to analyze epidemiological data on patients presenting to the ED because of dyspnea at the Cantonal Hospital Baselland (KSBL) in Switzerland and to audit the assessment and management of the patients performed at the ED.

## 2. Materials and Methods

### 2.1. Study Design

This retrospective, observational study was conducted in a single-center setting at KSBL in Switzerland. The study protocol was reviewed and approved by the Ethics Committee of Northwest and Central Switzerland (ENKZ, BASEC Project-ID 2022-02004).

### 2.2. Patient Population

The study included patients who presented to the ED of KSBL with dyspnea as their primary symptom, were admitted to the internal medicine ward for at least one night, and were discharged between January 2022 and December 2022. Patients who had declined to provide general consent for the use of health-related data and samples for research purposes were not included in the analysis.

### 2.3. Patient Selection and Data Collection Process

Data collection was performed manually by a physician using all available electronic patient records within the KISIM server used at KSBL. Data management was carried out using Research Electronic Data Capture (REDCap^®^), a secure web-based platform designed to facilitate data capture for research purposes [30,31]. To ensure data accuracy, a second study physician reviewed a subset of the data. Additionally, plausibility checks were performed by re-evaluating the five highest and five lowest outliers.

**Figure 1:**
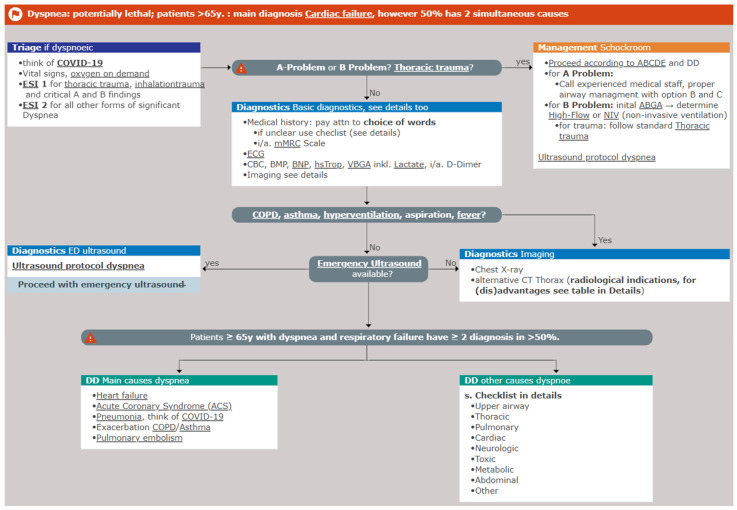
MedStandards dyspnea algorithm.

As main comparison for the audit we used the medStandards algorithm suggested by Professor Roland Bingisser and his team at University Hospital Basel, which is displayed in Figure 1 [32]. This algorithm is based mainly on international guidelines of diseases presenting often with dyspnea, such as acute coronary syndrome (ACS) [33], pulmonary embolism (PE) [34], and heart failure [35]. Furthermore, it is based on studies about acute respiratory failure management [23,36,37], clinical examinations [38], and usefulness of dyspnea index scales [39].

## 3. Results

### 3.1. Patient Characteristics

The patient characteristics are summarized in Table 1. The median age of the patients at their presentation to the ED was 76 years (IQR 66–84). Male patients constituted 57% of the cohort. Most of the patients who arrived at the ED were self-referred (40.2%), while 35.0% arrived in an ambulance. A total of 94.3% had at least one comorbidity. A total of 87.8% of these comorbidities were cardiovascular diseases with arterial hypertension being the most frequent (81.5%), followed by heart failure (39.2%), dyslipidemia (38.8%), arrhythmias (37.9%), and coronary heart disease (35.7%). Diabetes was present in 30.2% of patients with comorbidities. Respiratory diseases were reported in 55.4% of the cases, with COPD (38.4%), a history of pneumonia (22.8%), a history of COVID-19 (21.2%), and obstructive sleep apnea (20.5%) being the most frequent ones. Smoking status was reported in only 41.4% of the cases. Only three patients had a history of neuromuscular disorders. When reported, most of the patients were either former smokers (45.2%) or current smokers (44.3%). Regarding these patients, pack years were reported in 75.1% of the cases with a median value of 45 (IQR 60–30). The body mass index (BMI) was reported in 37.8% of the patients with the majority being obese (median 27.5 kg/m2, IQR 23.75–32.2). Only 8.5% of the patients reported having allergies.

### 3.2. Clinical Characteristics

The patients’ clinical characteristics at admission are shown in Table 2. Acute dyspnea (defined as a new onset of dyspnea in the past 14 days) was observed in 84.2% of the patients, while 14.1% had acute-on-chronic dyspnea (defined as a worsening of pre-existing dyspnea in the past 14 days). Only 0.5% had stable chronic dyspnea, and in 1.2% of the cases, the onset of the symptom was not specified. In patients with associated symptoms (78.5%), 52.3% experienced a cough, 23.8% experienced sputum production, and 23.5% experienced chest pain. Remarkably, in only 14.8% of the cases, a description of the patient’s subjective dyspnea sensation was reported. The most frequent description was chest tightness or constriction (11.4%). Regarding the description of the effort triggering dyspnea, in only slightly more than one-third of the cases (36.2%) was the description sufficient to categorize the dyspnea within the NYHA scale. Most of these patients were describing a dyspnea classifiable as NYHA III (19.3%). In only 7.8% of the cases was the dyspnea explicitly assessed by the physician using a dyspnea index scale. The NYHA scale was used in 100% of these cases. Regarding vital signs, the systolic blood pressure was reported in 93.8%, the diastolic blood pressure in 93.3%, the heart rate in 93.8%, the respiratory rate in 61.4%, the oxygen saturation in 96.1%, the amount of oxygen supplementation in 95.8%, and the body temperature in 86.3% of the cases. Among patients with reported relevant cardiopulmonary clinical signs, 82.9% presented with peripheral edema and 36.6% with jugular vein distension. Lung percussion was reported in only 2.6% of the cases, while lung auscultation was reported in 94.4% of the patients. Crackles were found in 35.3% of patients, and 33.2% had normal lung auscultation. Heart auscultation was reported less frequently (85.3%), with 72.5% having normal examinations.

### 3.3. Diagnostics and Therapy

Major diagnostic steps are described in Table 3. Instrumental diagnostics were performed in 96.8% of cases, with electrocardiogram (ECG) being the most common (90.3%), followed by chest radiography (40.5%) and thoracic computer tomography (37.0%). Ultrasounds were performed very rarely (4.4% received an echocardiography and 3.4% a lung ultrasound). A basic metabolic panel with a complete blood count was performed very often but not in all patients with a range from 86.9% (with a differential blood count test) to 97.7% (with or without a differential blood count test). TSH levels were measured in 86.7% of the patients, while an arterial blood gas analysis was performed in 23.6% and a venous blood gas analysis was performed in 12.4% of the cases.

### 3.4. Final Diagnosis and Outcome

Final diagnoses and outcomes are presented in Table 4. Almost every patient had a final diagnosis explaining the cause of their dyspnea (95.7%). The most frequent diagnosis was decompensated heart failure (28.4%), followed by pneumonia (26.4%) and COVID-19 (17.0%). After a median length of hospital stay of 7 days (IQR 4–10), most of the patients were discharged home (74.7%), while 5.8% died during their hospitalization. Ambulatory rehabilitation was prescribed for 10.9% of the patients. A total of 16.1% died within 12 months after their discharge, while during the same period, 37.4% of the patients presented again to an ED and were hospitalized. Most of them (46.8%) presented for a different reason compared to the previous hospitalization. Nonetheless, 19.5% had a relapse of the previous diagnosis within 12 months.

## 4. Discussion

Our study provides a comprehensive evaluation of the clinical management of patients presenting with dyspnea to the ED of KSBL in Switzerland. We found several significant gaps in the adherence to the medStandards guidelines, highlighting areas for improvement in the assessment and management of dyspnea cases. The main findings are as follows: vital signs were reported very often except for the respiratory rate and the body temperature. No patients with neuromuscular disorders were admitted to the shock room. The patient’s smoking status was not reported in most cases. The same can be said about the dyspnea sensation description, the physical effort triggering the dyspnea, and the use of a dyspnea index scale to assess the dyspnea’s intensity. Finally, an emergency ultrasound should be performed much more often than it is.

Regarding demographic characteristics, the patients’ median age (76 years) was higher than the one examined in others epidemiologic studies by Kelly A.M. et al. [21] and Laribi S. et al. [22] (67 years and 68 years, respectively). These are two international, multicenter, prospective, observational, cohort studies, designed to evaluate the epidemiology and outcomes of patients presenting to the ED with dyspnea as their main complaint. The male sex was also more present in our sample (57% vs. 49.1% and 49.0%). The prevalence of comorbidities, however, was similar, with hypertension being by far the most frequent one. Nonetheless, patients with asthma were much rarer in our group (8.1% vs. 22.6% and 20.6%). This may be due to the high median age of our sample and the predominance of the male sex. In fact, adult asthma is less prevalent than childhood asthma and has a female predominance [40,41,42].

Analyzing the final diagnosis, our results showed some similarities but also some differences to the epidemiologic studies by Kelly A.M. et al. [21] and Laribi S. et al. [22]. Considering all type of respiratory tract infections as a single group, we have very similar results. These infections account for the most frequent diagnosis, followed by decompensated heart failure and COPD exacerbation. However, exacerbated asthma was not a frequent diagnosis in our study (3.6% vs. 12.7% and 10.5%). This may be due to the same reasons for the lower rate of asthma as a comorbidity discussed above. In our study, more patients received an ACS (11.5% vs. 3.1%), arrhythmia (9.1% vs. 2.6%), or PE diagnosis (6.5% vs. 1.2%). In the literature, it is reported that the prevalence of these diseases increases with age and for the male sex [43,44,45,46,47]. With our sample having a higher median age and a male predominance compared to the other studies, this could explain the difference in results. Regarding the outcome, our study showed a slightly longer median length of hospital stay (7 days vs. 5 days) with a very similar in-hospital death rate (5.8% vs. 5–6%).

### 4.1. Comparison to the Guidelines: Narrative Discussion

- The following vital signs should be collected in all patients: blood pressure, heart rate, respiratory rate, oxygen saturation, and body temperature.

Our study revealed that vital signs were generally well documented, with systolic and diastolic blood pressure, heart rate, and oxygen saturation recorded in over 93% of cases. However, respiratory rate and body temperature were less frequently reported (in only 61.4% and 86.3% of the cases, respectively). In comparison, the studies by Kelly A.M. et al. [21] and Laribi S. et al. [22] had reported respiratory rates for 96.9% and 89.8% of the patients, respectively. This gap indicates a significant area for improvement, as the respiratory rate is a critical parameter in the assessment of dyspnea and is emphasized in the medStandards.

In fact, the respiratory rate is a fundamental vital sign that should be used to monitor every patient in the ED [48]. Monitoring a patient’s respiratory rate is an essential early indicator of systemic illness and a predictor of adverse outcomes, including cardiac arrest and the need for ICU admission or transfer [49,50,51,52,53,54]. Despite its importance as an early predictor of clinical deterioration, it remains the least frequently measured and accurately documented vital sign [55,56]. Several studies, including those by Badawy et al. and Ludikhuize et al. [57,58], have highlighted that the respiratory rate is frequently neglected or inaccurately recorded in clinical practice, indicating a systemic issue in monitoring the vital signs of hospitalized patients.

Several factors could contribute to the underreporting of the respiratory rate of ED patients [59,60], such as inadequate knowledge, reliance on subjective assessments, and rationalized judgments based on experience [61,62,63]. Moreover, clinicians might prioritize other assessments due to limited resources. Consequently, the recording of the respiratory rate, which typically necessitates manual counting, may be inadvertently neglected.

- Patients with known neuromuscular disorders should be admitted to the shock room first.

Our study population included only three patients with a neuromuscular disease (one with MG, one with DMD, and one with ALS), and none of them were admitted to the shock room initially. While the number of affected patients is low, this highlights a potential oversight in protocol adherence for this high-risk subgroup.

However, we consider the medStandards algorithm in this regard too vague. In fact, the algorithm does not provide a specific list of neuromuscular disorders to consider, which leaves room for interpretation; moreover, admitting patients with neuromuscular disorders to the shock room may not always be appropriate, e.g., in palliative care situations. The literature emphasizes the importance of aligning medical interventions with patient preferences, especially in advanced stages of neuromuscular diseases in which the focus often shifts to comfort care and palliation [64].

To improve the medStandards recommendations, we suggest specifying which neuromuscular disorders require shock room admission and under what circumstances. Additionally, the guidelines should include considerations for patient-centered care, particularly for those in palliative situations.

- All patients should receive the following basic diagnostics: medical history (with a description of the feeling of and situations triggering the dyspnea, a description of whether the dynamic was acute, acute-on-chronic, or chronic, a categorization of the intensity within a dyspnea index scale, and an assessment of smoking status), clinical examination (including percussion and auscultation), laboratory values (complete blood count, basic metabolic panel, venous blood gas analysis, and TSH test), and ECG.

The reporting of the dyspnea sensation (14.8%), the effort triggering the dyspnea (36.2%), and the use of an index dyspnea scale (7.8%) was very scarce and performed only with the NYHA scale, which is significantly below the recommended standard, more so considering that 32.4% of the patients had a history of heart failure. Interestingly, one patient was described as having NYHA I, which is per its definition the absence of dyspnea.

Challenges include time constraints and variability in patient descriptions. The precise documentation of dyspnea descriptions can help to differentiate between various causes of dyspnea, such as cardiac versus pulmonary etiologies, and aid in risk stratification by identifying potential comorbidities that may influence treatment choices. Furthermore, accurate documentation enables us to improve the diagnostic accuracy as well as follow-up and chronic disease management (Santus et al. [65], Hashimi et al. [66], and Azeemuddin et al. [67]).

Smoking status was reported in less than half of the patients (41.4%). When it was reported, most of the patients were either former smokers or current smokers, hinting that life-long non-smokers may be frequent in the group that did not report smoking. Given the well-established link between smoking and numerous respiratory and cardiovascular diseases [68], this is a significant oversight. The accurate documentation of smoking status is crucial as it can influence both diagnosis and management strategies, including the need for smoking cessation interventions, which are critical for improving patient outcomes [69,70,71]. In our previous published audits assessing the management of community-acquired pneumonia (CAP) [72] and asthma [73], we also found that smoking status was poorly reported (52% and 58.7%, respectively). These findings align with our results and underscore a common issue in emergency care settings.

Focusing on clinical examination, the medStandards highlights the importance of performing lung percussion and cardiopulmonary auscultation in all patients. In our study, only 2.6% of the patients received lung percussion, while 94.4% received lung auscultation and 85.3% received heart auscultation. Similar results were reported in the epidemiological study by Kelly A.M. et al. [21], with 95% of the patients receiving chest auscultation, while data regarding lung percussion were not included in the study. As it is still considered a cornerstone of the cardiopulmonary physical examination [74] and it yields important diagnostic information [75], lung percussion should be performed much more often.

Regarding laboratory investigations, all the values recommended by the medStandards were frequently collected (ranging from 86.9% to 97.7%), except for the blood gas analysis (with 34.0% being either arterial or venous analyses). In this regard, as already examined in the introduction, there are conflicting data in the literature about performing a blood gas analysis for all patients presenting to the ED. Hence, we think that this low rate of blood gas analysis measurements at KSBL may be due to the absence of a consensus, reserving this examination for specific groups of patients (e.g., critically ill or unstable). Compared to the epidemiological study by Kelly A.M. et al. [21], we had very similar results except for CRP (97.3% vs. 30.7%), procalcitonin (13.7% vs. 0.9%), NT-pro-BNP (64.8% vs. 8.3%), and D-dimers (36.2% vs. 3.5%), which were reported much more often in our study. The higher rate of the CRP measurements may be explained by the use of block laboratory panels, which often directly include CRP. However, the medStandards and the literature [76,77,78] do not advise measuring CRP in every patient presenting to the ED.

Our data show a high compliance rate regarding ECG, which was performed in 90.3% of the patients. This high adherence to the guidelines may be due the ECG’s widely known ability to rapidly identify critical cardiac conditions, such as ACS, arrhythmias, signs of PE, and signs of heart failure [79].

- The first imaging test should be an emergency ultrasound except in patients with known or suspected COPD, asthma, hyperventilation, aspiration, or fever, for whom CRX/CT imaging is more appropriate.

Our data reveal a high overall rate of instrumental diagnostics (96.8%), with the most frequently performed tests being ECGs (90.3%), CRXs (40.5%), and thoracic CTs (37.0%). However, the use of ultrasounds was notably rare, with only 4.4% of patients receiving an echocardiography and 3.4% a lung ultrasound.

Point-of-care ultrasound (POCUS) is an innovative, non-invasive, radiation-free bedside diagnostic tool that provides real-time information and is transforming clinical care globally [80,81,82]. Studies have shown that while POCUS can be highly effective in diagnosing various causes of dyspnea [83], its adoption in EDs is hindered by a lack of trained personnel and established protocols [84]. The accessibility and familiarity of CRX and CT scans may lead clinicians to default to these modalities instead of ultrasounds.

Our study revealed different results compared to the epidemiological study by Kelly A.M. et al. [21]. Although chest x-rays were commonly performed in our study, they were utilized even more frequently in those studies (40.5% vs. 86.1%). While lung ultrasounds were rarely conducted in our study, they were employed more in those studies (3.4% vs. 0.6%). Thus, despite our study’s relatively better results, there is a need to increase the frequency of ultrasound use at the ED at KSBL while reducing the reliance on x-rays, particularly in cases in which an ultrasound could provide valuable diagnostic insights.

Adhering to the recommended imaging guidelines is crucial in terms of diagnostic efficiency, radiation exposure, and cost-effectiveness [83,85,86].

### 4.2. Study Limitations

Despite the large sample size, our study is limited by its retrospective and descriptive design, which restricts our ability to infer causality between guideline adherence and patient outcomes. Furthermore, our analysis was conducted in a single university teaching hospital, focusing exclusively on patients admitted to the internal medicine department in 2022. This may limit the generalizability of our findings, as dyspnea cases can also be managed in other departments, such as surgery (e.g., patients with pneumothorax or rib fractures), intensive care, or cardiology. Future studies should expand the research scope to multiple hospitals and specialties to improve its external validity.

Although the potential limitation of a retrospective study is accurately collecting data, another physician conducted a sample check by recollecting the data. Upon comparing the results, the data proved to be reliable and accurate. Another limitation is that when the patient record did not provide a numeric value of the vital sign, we considered it as not reported, even if there was some written reporting, such as “normotonic”. This means that our interpretation of the patient records may be slightly different if others performed it. In general, we also need to explain that “not reported” only means that we could not find a clear reporting in the patient records. Hence, it could be that a certain investigation was performed but not reported, which may have led to the underestimation of the actual guideline adherence. Finally, our study highlights gaps in history taking and diagnostic assessments, but without prospective validation, we cannot determine the direct impact of these gaps on clinical outcomes. Moving forward, well-powered prospective studies comparing guideline-directed care with usual clinical practice will be essential to establish evidence-based standards for managing dyspnea in the ED. Additionally, future research should focus on assessing guideline adherence in treatment decisions to further optimize patient management and outcomes.

## 5. Conclusions

This study provides valuable characteristics and clinical information on patients presenting to the ED because of dyspnea. Based on the medStandards of the University Hospital of Basel, our study showed that patients presenting to the ED in KSBL in 2022 because of dyspnea were not always correctly assessed and managed. In conclusion, our study showed the following main findings: vital signs were reported very often except for the respiratory rate and the body temperature. No patients with neuromuscular disorders were admitted to the shock room. The patient’s smoking status was not reported in most cases. The same can be said about the dyspnea sensation description, the physical effort triggering dyspnea, and the use of a dyspnea index scale to assess the dyspnea’s intensity. Finally, an emergency ultrasound should be performed much more often than it is. In particular, the respiratory rate measurement, a complete and detailed anamnesis collection, and the use of an emergency ultrasound could be improved. As dyspnea is a frequent reason for ED presentation, there is an urgent need for its amelioration. This could be accelerated by providing clear international guidelines for the assessment and management of patients with dyspnea coming to the ED. We consider our study a starting point for future prospective studies, which could contribute to expedite the creation of international guidelines.

To enhance the management of dyspnea in the ED, we propose the following recommendations:

1. Standardized Training and Protocols: Implement comprehensive training for ED staff on the importance of complete vital sign documentation, including respiratory rate and body temperature, and the systematic use of dyspnea assessment scales;
2. Refined Guidelines for Neuromuscular Disorders: Update the medStandards to specify which neuromuscular disorders warrant shock room admission and include criteria for exceptions, particularly in palliative care contexts, allowing for individualized patient care;
3. Enhanced Use of Ultrasound: Promote the use of emergency ultrasound through training and resource allocation, emphasizing its role in rapid and accurate diagnosis.
4. Quality Improvement Initiatives: Regularly audit clinical practices and provide feedback to ED staff to ensure adherence to guidelines and identify areas for ongoing improvement.

By addressing these gaps, we can improve the quality of care for patients presenting with dyspnea, leading to better outcomes and the more efficient use of ED resources.

## Figures and Tables

**Table 1:** Patient characteristics (*n* = 823).

Demographic, *n* (%)	823 (100.0)
Age at admission, median (IQR) in years; range	76 (66–84); (15–99)
Gender (male), *n* (%)	469 (57)
**Presentation modality, *n* (%)**	**823 (100.0)**
Self-referred, *n* (%)	331 (40.2)
Ambulance, *n* (%)	288 (35.0)
From general practitioner (GP), *n* (%)	140 (17.0)
From doctor in KSBL, *n* (%)	33 (4.0)
From another doctor, *n* (%)	22 (2.7)
From another hospital, *n* (%)	5 (0.6)
From psychiatry, *n* (%)	4 (0.5)
**Comorbidities, *n* (%)**	**776 (94.3)**
Cardiovascular diseases	681 (87.8)
Heart failure, *n* (%)	267 (39.2)
Coronary heart disease, *n* (%)	243 (35.7)
Arterial hypertension, *n* (%)	555 (81.5)
Dyslipidemia, *n* (%)	264 (38.8)
Arrhythmias, *n* (%)	258 (37.9)
Peripheral artery disease, *n* (%)	87 (12.8)
Cerebrovascular diseases, *n* (%)	98 (14.4)
Other, *n* (%)	66 (9.7)
Respiratory diseases	430 (55.4)
COPD, *n* (%)	165 (38.4)
Asthma, *n* (%)	67 (15.6)
History of pneumonia, *n* (%)	98 (22.8)
History of pulmonary embolism, *n* (%)	71 (16.5)
History of COVID-19, *n* (%)	91 (21.2)
Active lung cancer, *n* (%)	25 (5.8)
Healed lung cancer, *n* (%)	8 (1.9)
OSA, *n* (%)	88 (20.5)
Other, *n* (%)	50 (11.6)
Diabetes, *n* (%)	234 (30.2)
Anxiety, *n* (%)	33 (4.3)
Depression, *n* (%)	89 (11.5)
Neuromuscular diseases *, *n* (%)	3 (0.4)
Non-pulmonary tumors, *n* (%)	170 (22.0)
**Risk Factors**	
Reported smoking status, *n* (%)	341 (41.4)
Never smoked, *n* (%)	36 (10.6)
Former smoker, *n* (%)	154 (45.2)
Reported pack years, *n* (%)	109 (70.8)
Current smoker, *n* (%)	151 (44.3)
Reported pack years, *n* (%)	120 (79.5)
Median pack years (IQR)	45 (60–30)
BMI, *n* (%); median in kg/m^2^ (IQR)	311 (37.8); 27.5 (23.75–32.2)
Patients with allergies, *n* (%)	70 (8.5)

OSA: obstructive sleep apnea, BMI; body mass index, IQR: interquartile range, KSBL: Cantonal Hospital Baselland; * Including only amyotrophic lateral sclerosis, myasthenia gravis, Guillain–Barré syndrome, and Duchenne muscular dystrophy.

**Table 2:** Clinical variables.

	All (*n* = 823)
**Dyspnea onset**	
Acute, *n* (%)	693 (84.2)
Acute-on-chronic, *n* (%)	116 (14.1)
Chronic, *n* (%)	4 (0.5)
Not specified, *n* (%)	10 (1.2)
**Associated symptoms, *n* (%)**	**646 (78.5)**
Cough, *n* (%)	338 (52.3)
Sputum production, *n* (%)	154 (23.8)
Nasal congestion, *n* (%)	24 (3.7)
Chest pain, *n* (%)	152 (23.5)
Peripheral edema, *n* (%)	59 (9.1)
Calf pain, *n* (%)	9 (1.4)
Fever, *n* (%)	117 (18.1)
Others, *n* (%)	277 (42.9)
**Dyspnea sensation**	
Increased work/effort, *n* (%)	7 (0.9)
Chest tightness, *n* (%)	94 (11.4)
Air hunger, *n* (%)	1 (0.1)
Shallow breathing, *n* (%)	2 (0.2)
Suffocating, *n* (%)	9 (1.1)
Heavy breathing, *n* (%)	5 (0.6)
Other, *n* (%)	4 (0.5)
Not reported, *n* (%)	701 (85.2)
**Effort triggering dyspnea**	
Intense exercise, *n* (%)	52 (6.3)
Regular activities, *n* (%)	159 (19.3)
Rest, *n* (%)	87 (10.6)
Not reported, *n* (%)	525 (63.8)
**Dyspnea index scale, *n* (%)**	**64 (7.8)**
NYHA scale, *n* (%)	64 (100.0)
NYHA I	1 (1.6)
NYHA II	13 (20.3)
NYHA III	25 (39.1)
NYHA IV	25 (39.1)
Other scale, *n* (%)	0 (0.0)
**Vital signs**	
Systolic BP, *n* (%); median in mmHg (IQR)	772 (93.8); 135 (152–118)
Diastolic BP, *n* (%); median in mmHg (IQR)	768 (93.3); 78.5 (89–69)
Heart rate, *n* (%); median in bpm (IQR)	772 (93.8); 86 (103–74)
Respiratory rate, *n* (%); median in brpm (IQR)	505 (61.4); 22 (26–18)
Oxygen saturation, *n* (%); median in % (IQR)	791 (96.1); 94 (96–91)
Oxygen supplementation reported, *n* (%)	788 (95.8)
Patients with supplementation, *n* (%)	235 (29.8)
Median in L/min (IQR)	3 (5–2)
Body temperature, *n* (%); median in °C (IQR)	710 (86.3); 36.8 (37.6–36.4)
**Clinical signs**	
Cyanosis, *n* (%)	2 (0.2)
Peripheral edema, *n* (%)	238 (28.9)
Calf compression pain, *n* (%)	7 (0.9)
Jugular vein distension, *n* (%)	105 (12.8)
Use of accessory respiratory muscles, *n* (%)	11 (1.3)
Changes in mental status, *n* (%)	19 (2.3)
Finger clubbing, *n* (%)	1 (0.1)
**Physical examination**	
Lung percussion, *n* (%)	21 (2.6)
Normal, *n* (%)	6 (28.6)
Reduced sound, *n* (%)	13 (61.9)
Increased sound, *n* (%)	2 (8.5)
Lung auscultation, *n* (%)	777 (94.4)
Normal, *n* (%)	258 (33.2)
Stridor, *n* (%)	6 (0.8)
Wheeze, *n* (%)	173 (22.3)
Rhonchi, *n* (%)	75 (9.7)
Crackles/rales, *n* (%)	274 (35.3)
Reduced/missing sounds, *n* (%)	177 (22.8)
Other, *n* (%)	10 (1.3)
Heart auscultation, *n* (%)	702 (85.3)
Normal, *n* (%)	509 (72.5)
Third sound, *n* (%)	0 (0.0)
Fourth sound, *n* (%)	0 (0.0)
Systolic murmur, *n* (%)	89 (12.7)
Diastolic murmur, *n* (%)	1 (0.1)
Other, *n* (%)	119 (17)

BP: blood pressure, NYHA: New York Heart Association, IQR: interquartile range.

**Table 3:** Major investigations.

	All (*n* = 823)
**Instrumental diagnostics, *n* (%)**	**797 (96.8)**
ECG, *n* (%)	720 (90.3)
Echocardiography, *n* (%)	35 (4.4)
Lung ultrasound, *n* (%)	27 (3.4)
CRX, *n* (%)	323 (40.5)
Thoracic CT, *n* (%)	295 (37.0)
Coronarography, *n* (%)	28 (3.5)
Other, *n* (%)	10 (1.3)
Patients who should receive ultrasound first *, *n*	337
CRX or thoracic CT, *n* (%)	200 (59.4)
Emergency ultrasound, *n* (%)	34 (10.1)
Patients who should receive CRX/CT **, *n*	486
CRX or thoracic CT, *n* (%)	402 (82.7)
Emergency ultrasound, *n* (%)	22 (4.5)
**Laboratory parameters, *n* (%)**	**815 (99.0)**
CRP, *n* (%); median in mg/L (IQR)	801 (98.3); 19 (83–4)
Procalcitonin, *n* (%); median in ng/mL (IQR)	113 (13.9); 0.08 (0.18–0.05)
Troponin T hs, *n* (%); median in ng/L (IQR)	330 (40.5); 34 (69.75–19.83)
NT-pro-BNP, *n* (%); median in ng/L (IQR)	533 (65.4); 1′709 (5′339–396)
TSH, *n* (%); median in mU/L (IQR)	707 (86.8); 1.80 (2.94–1.08)
Leucocytes, *n* (%); median in × 10^9^/L (IQR)	797 (97.8); 9.1 (12.2–7.0)
Hemoglobin, *n* (%); median in g/L (IQR)	797 (97.8); 133 (146–117)
D-dimers, *n* (%); median in μg/mL (IQR)	298 (36.6); 1.04 (2.06–0.57)
Arterial blood gas analysis, *n* (%)	192 (23.6)
Venous blood gas analysis, *n* (%)	101 (12.4)
SARS-CoV-2 PCR, *n* (%)	325 (39.9)
Positive, *n* (%)	49 (15.1)
Multiplex respiratory panel PCR, *n* (%)	316 (38.4)
Positive, *n* (%)	101 (32.0)
Urine antigen testing, *n* (%)	242 (29.7%)
Blood cultures, *n* (%)	210 (24.7)

* Defined as patients without known or suspected COPD, asthma, hyperventilation (approximated as tachypnea of >22 breaths per minute), aspiration, or fever (defined as reported as symptom by the patient or as a measured body temperature of >37.5 °C). ** Defined as patients with known or suspected COPD, asthma, hyperventilation (approximated as tachypnea of >22 breaths per minute), aspiration, or fever (defined as reported as symptom by the patient or as a measured body temperature of >37.5 °C). ECG: Electrocardiogram. CRX: Chest X-ray, CT: Computed tomography, CRP: C-reactive protein, NT-pro-BNP: N-terminal pro B-type natriuretic peptide, D-dimers: D-dimer, TSH: Thyroid stimulating hormone, SARS-CoV-2: Severe Acute Respiratory Syndrome Coronavirus 2, PCR: Polymerase chain reaction, Multiplex respiratory panel PCR: Multiplex respiratory pathogen panel polymerase chain reaction.

**Table 4:** Final diagnosis and outcome.

	All (*n* = 823)
**Final diagnosis, *n* (%)**	**788 (95.7)**
Pneumonia, *n* (%)	208 (26.4)
Pulmonary embolism, *n* (%)	51 (6.5)
Asthma exacerbation, *n* (%)	28 (3.6)
COPD exacerbation, *n* (%)	97 (12.3)
COVID-19, *n* (%)	134 (17.0)
Other respiratory tract infection, *n* (%)	51 (6.5)
Acute coronary syndrome, *n* (%)	91 (11.5)
Decompensated heart failure, *n* (%)	224 (28.4)
Arrhythmia, *n* (%)	72 (9.1)
Anaphylactic reaction, *n* (%)	14 (1.8)
Panic attack/anxiety, *n* (%)	9 (1.1)
Other, *n* (%)	127 (16.1)
**Outcome, *n* (%)**	**823 (100.0)**
Length of stay in days, median (IQR)	7 (4–10)
Discharged home, *n* (%)	615 (74.7)
Outpatient rehabilitation, *n* (%)	67 (10.9)
Other hospital, *n* (%)	30 (3.6)
Care facility, *n* (%)	63 (7.7)
Rehabilitation hospital, *n* (%)	67 (8.1)
Death in hospital, *n* (%)	48 (5.8)
Death within 12 months, *n* (%)	125 (16.1) *
Rehospitalization within 12 months, *n* (%)	308 (39.7) *
Same diagnosis, *n* (%)	11 (3.6)
Relapse, *n* (%)	60 (19.5)
Cardiovascular disease, *n* (%)	33 (10.7)
Respiratory disease, *n* (%)	60 (19.5)
Other, *n* (%)	144 (46.8)

* Percentage calculated excluding patients who died during their hospitalization. Rehospitalization considered only inpatients presenting to the ED of KSBL first.

## Data Availability

All data generated during this study were analyzed and the results were included in this article. The data presented in this study are available on reasonable request from the corresponding author. The data are not publicly available due to restrictions on data privacy.

## References

[1] Elliott M.W., Adams L., Cockcroft A., MacRae K.D., Murphy K., Guz A. (1991). The Language of Breathlessness: Use of Verbal Descriptors by Patients with Cardiopulmonary Disease. Am. Rev. Respir. Dis..

[2] Simon P.M., Schwartzstein R.M., Weiss J.W., Fencl V., Teghtsoonian M., Weinberger S.E. (1990). Distinguishable Types of Dyspnea in Patients with Shortness of Breath. Am. Rev. Respir. Dis..

[3] Parshall M.B., Schwartzstein R.M., Adams L., Banzett R.B., Manning H.L., Bourbeau J., Calverley P.M., Gift A.G., Harver A., Lareau S.C. (2012). An Official American Thoracic Society Statement: Update on the Mechanisms, Assessment, and Management of Dyspnea. Am. J. Respir. Crit. Care Med..

[4] Campbell M.L. (2008). Psychometric testing of a respiratory distress observation scale. J. Palliat. Med..

[5] Campbell M.L. (2008). Respiratory distress: A model of responses and behaviors to an asphyxial threat for patients who are unable to self-report. Heart Lung.

[6] Lush M.T., Janson-Bjerklie S., Carrieri V.K., Lovejoy N. (1988). Dyspnea in the ventilator-assisted patient. Heart Lung.

[7] Schwartzstein R.M., Gold D.R. (2009). Dyspnea in overweight children: Is it asthma?. J. Allergy Clin. Immunol..

[8] Campbell M.L., Templin T., Walch J. (2010). A Respiratory Distress Observation Scale for patients unable to self-report dyspnea. J. Palliat. Med..

[9] Desbiens N.A., Mueller-Rizner N., Connors A.F., Wenger N.S. (1997). The relationship of nausea and dyspnea to pain in seriously ill patients. Pain.

[10] Hammond E.C. (1964). Some Preliminary Findings on Physical Complaints from a Prospective Study of 1,064,004 Men and Women. Am. J. Public Health Nations Health.

[11] Kroenke K., Arrington M.E., Mangelsdorff A.D. (1990). The prevalence of symptoms in medical outpatients and the adequacy of therapy. Arch. Intern. Med..

[12] Currow D.C., Plummer J.L., Crockett A., Abernethy A.P. (2009). A community population survey of prevalence and severity of dyspnea in adults. J. Pain. Symptom Manag..

[13] Grønseth R., Vollmer W.M., Hardie J.A., Ólafsdóttir I.S., Lamprecht B., Buist A.S., Gnatiuc L., Gulsvik A., Johannessen A., Enright P. (2014). Predictors of dyspnoea prevalence: Results from the BOLD study. Eur. Respir. J..

[14] Ho S.F., O’Mahony M.S., Steward J.A., Breay P., Buchalter M., Burr M.L. (2001). Dyspnoea and quality of life in older people at home. Age Ageing.

[15] Perri M.G., Anton S.D., Durning P.E., Ketterson T.U., Sydeman S.J., Berlant N.E., Kanasky W.F., Newton R.L., Limacher M.C., Martin A.D. (2002). Adherence to exercise prescriptions: Effects of prescribing moderate versus higher levels of intensity and frequency. Health Psychol..

[16] Hayton C., Clark A., Olive S., Browne P., Galey P., Knights E., Staunton L., Jones A., Coombes E., Wilson A.M. (2013). Barriers to pulmonary rehabilitation: Characteristics that predict patient attendance and adherence. Respir. Med..

[17] Milos R.I., Bartha C., Röhrich S., Heidinger B.H., Prayer F., Beer L., Wassipaul C., Kifjak D., Watzenboeck M.L., Pochepnia S. (2023). Imaging in patients with acute dyspnea when cardiac or pulmonary origin is suspected. BJR Open.

[18] Logeart D., Saudubray C., Beyne P., Thabut G., Ennezat P.V., Chavelas C., Zanker C., Bouvier E., Solal A.C. (2002). Comparative value of Doppler echocardiography and B-type natriuretic peptide assay in the etiologic diagnosis of acute dyspnea. J. Am. Coll. Cardiol..

[19] Solano J.P., Gomes B., Higginson I.J. (2006). A Comparison of Symptom Prevalence in Far Advanced Cancer, AIDS, Heart Disease, Chronic Obstructive Pulmonary Disease and Renal Disease. J. Pain Symptom Manag..

[20] Cairns C.K.K. (2021). National Hospital Ambulatory Medical Care Survey: 2021 Emergency Department Summary Tables. https://ftp.cdc.gov/pub/Health_Statistics/NCHS/Dataset_Documentation/NHAMCS/doc21-ed-508.pdf.

[21] Kelly A.M., Keijzers G., Klim S., Graham C.A., Craig S., Kuan W.S., Jones P., Holdgate A., Lawoko C., Laribi S. (2017). An Observational Study of Dyspnea in Emergency Departments: The Asia, Australia, and New Zealand Dyspnea in Emergency Departments Study (AANZDEM). Acad. Emerg. Med..

[22] Laribi S., Keijzers G., van Meer O., Klim S., Motiejunaite J., Kuan W.S., Body R., Jones P., Karamercan M., Craig S. (2019). AANZDEM and EURODEM study groups. Epidemiology of patients presenting with dyspnea to emergency departments in Europe and the Asia-Pacific region. Eur. J. Emerg. Med..

[23] Ray P., Birolleau S., Lefort Y., Becquemin M.H., Beigelman C., Isnard R., Teixeira A., Arthaud M., Riou B., Boddaert J. (2006). Acute respiratory failure in the elderly: Etiology, emergency diagnosis and prognosis. Crit. Care.

[24] Kelly A.-M., Keijzers G., Klim S., Craig S., Kuan W.S., Holdgate A., Graham C.A., Jones P., Laribi S. (2020). Epidemiology and outcome of older patients presenting with dyspnoea to emergency departments. Age Ageing.

[25] Sørensen S.F., Ovesen S.H., Lisby M., Mandau M.H., Thomsen I.K., Kirkegaard H. (2021). Predicting mortality and readmission based on chief complaint in emergency department patients: A cohort study. Trauma Surg. Acute Care Open.

[26] Safwenberg U., Terént A., Lind L. (2007). The Emergency Department presenting complaint as predictor of in-hospital fatality. Eur. J. Emerg. Med..

[27] Liteplo A.S., Marill K.A., Villen T., Miller R.M., Murray A.F., Croft P.E., Capp R., Noble V.E. (2009). Emergency Thoracic Ultrasound in the Differentiation of the Etiology of Shortness of Breath (ETUDES): Sonographic B-lines and N-terminal Pro-brain-type Natriuretic Peptide in Diagnosing Congestive Heart Failure. Acad. Emerg. Med..

[28] Nishimura K., Izumi T., Tsukino M., Oga T. (2002). Dyspnea Is a Better Predictor of 5-Year Survival Than Airway Obstruction in Patients With COPD. Chest.

[29] Abidov A., Rozanski A., Hachamovitch R., Hayes S.W., Aboul-Enein F., Cohen I., Friedman J.D., Germano G., Berman D.S. (2005). Prognostic Significance of Dyspnea in Patients Referred for Cardiac Stress Testing. N. Engl. J. Med..

[30] Harris P.A., Taylor R., Thielke R., Payne J., Gonzalez N., Conde J.G. (2009). Research electronic data capture (REDCap)—A metadata-driven methodology and workflow process for providing translational research informatics support. J. Biomed. Inform..

[31] Harris P.A., Taylor R., Minor B.L., Elliott V., Fernandez M., O′Neal L., McLeod L., Delacqua G., Delacqua F., Kirby J. (2019). The REDCap consortium: Building an international community of software platform partners. J. Biomed. Inform..

[32] Christiane R., Roland P.B., Gregory M. (2021). Dyspnoe/Hauptfolie. https://medstandards.com/medstandards-server/standard/57975?search=Bingisser%20.

[33] Roffi M., Patrono C., Collet J.P., Mueller C., Valgimigli M., Andreotti F., Bax J.J., Borger M.A., Brotons C., Chew D.P. (2016). 2015 ESC Guidelines for the management of acute coronary syndromes in patients presenting without persistent ST-segment elevation: Task Force for the Management of Acute Coronary Syndromes in Patients Presenting without Persistent ST-Segment Elevation of the European Society of Cardiology (ESC). Eur. Heart J..

[34] Konstantinides S.V., Torbicki A., Agnelli G., Danchin N., Fitzmaurice D., Galiè N., Gibbs J.S., Huisman M.V., Humbert M., Kucher N. (2014). 2014 ESC guidelines on the diagnosis and management of acute pulmonary embolism. Eur. Heart J..

[35] Ponikowski P., Voors A.A., Anker S.D., Bueno H., Cleland J.G.F., Coats A.J.S., Falk V., González-Juanatey J.R., Harjola V.P., Jankowska E.A. (2016). 2016 ESC Guidelines for the diagnosis and treatment of acute and chronic heart failure: The Task Force for the diagnosis and treatment of acute and chronic heart failure of the European Society of Cardiology (ESC)Developed with the special contribution of the Heart Failure Association (HFA) of the ESC. Eur. Heart J..

[36] Nee P.A., Al-Jubouri M.A., Gray A.J., O’Donnell C., Strong D. (2011). Critical care in the emergency department: Acute respiratory failure. Emerg. Med. J..

[37] Delerme S., Ray P. (2008). Acute respiratory failure in the elderly: Diagnosis and prognosis. Age Ageing.

[38] Spicknall K.E., Zirwas M.J., English J.C. (2005). Clubbing: An update on diagnosis, differential diagnosis, pathophysiology, and clinical relevance. J. Am. Acad. Dermatol..

[39] Bestall J.C., Paul E.A., Garrod R., Garnham R., Jones P.W., Wedzicha J.A. (1999). Usefulness of the Medical Research Council (MRC) dyspnoea scale as a measure of disability in patients with chronic obstructive pulmonary disease. Thorax.

[40] Dharmage S.C., Perret J.L., Custovic A. (2019). Epidemiology of Asthma in Children and Adults. Front. Pediatr..

[41] Trivedi M., Denton E. (2019). Asthma in Children and Adults-What Are the Differences and What Can They Tell us About Asthma?. Front. Pediatr..

[42] Chowdhury N.U., Guntur V.P., Newcomb D.C., Wechsler M.E. (2021). Sex and gender in asthma. Eur. Respir. Rev..

[43] Li S., Chaudhri K., Michail P., Gnanenthiran S.R. (2022). Acute coronary syndrome in older populations: Integrating evidence into clinical practice. Vessel. Plus.

[44] Mirza M., Strunets A., Shen W.K., Jahangir A. (2012). Mechanisms of arrhythmias and conduction disorders in older adults. Clin. Geriatr. Med..

[45] Curtis A.B., Karki R., Hattoum A., Sharma U.C. (2018). Arrhythmias in Patients ≥80 Years of Age: Pathophysiology, Management, and Outcomes. J. Am. Coll. Cardiol..

[46] Jarman A.F., Mumma B.E., Singh K.S., Nowadly C.D., Maughan B.C. (2021). Crucial considerations: Sex differences in the epidemiology, diagnosis, treatment, and outcomes of acute pulmonary embolism in non-pregnant adult patients. J. Am. Coll. Emerg. Physicians Open.

[47] Castelli R., Bergamaschini L.C., Sailis P., Pantaleo G., Porro F. (2009). The impact of an aging population on the diagnosis of pulmonary embolism: Comparison of young and elderly patients. Clin. Appl. Thromb. Hemost..

[48] Lee J.H., Nathanson L.A., Burke R.C., Anthony B.W., Shapiro N.I., Dagan A.S. (2024). Assessment of respiratory rate monitoring in the emergency department. J. Am. Coll. Emerg. Physicians Open.

[49] Armitage M., Eddleston J., Stokes T. (2007). Recognising and responding to acute illness in adults in hospital: Summary of NICE guidance. BMJ.

[50] Cuthbertson B.H., Boroujerdi M., McKie L., Aucott L., Prescott G. (2007). Can physiological variables and early warning scoring systems allow early recognition of the deteriorating surgical patient?. Crit. Care Med..

[51] Hong W., Earnest A., Sultana P., Koh Z., Shahidah N., Ong M.E. (2013). How accurate are vital signs in predicting clinical outcomes in critically ill emergency department patients. Eur. J. Emerg. Med..

[52] Subbe C.P., Davies R.G., Williams E., Rutherford P., Gemmell L. (2003). Effect of introducing the Modified Early Warning score on clinical outcomes, cardio-pulmonary arrests and intensive care utilisation in acute medical admissions. Anaesthesia.

[53] Buist M., Bernard S., Nguyen T.V., Moore G., Anderson J. (2004). Association between clinically abnormal observations and subsequent in-hospital mortality: A prospective study. Resuscitation.

[54] Loughlin P.C., Sebat F., Kellett J.G. (2018). Respiratory Rate: The Forgotten Vital Sign—Make It Count!. Jt. Comm. J. Qual. Patient Saf..

[55] Hill A., Kelly E., Horswill M.S., Watson M.O. (2018). The effects of awareness and count duration on adult respiratory rate measurements: An experimental study. J. Clin. Nurs..

[56] Takayama A., Takeshima T., Nagamine T. (2022). Factors associated with the frequency of respiratory rate measurement by hospital nurses: A multicentre cross-sectional study. Br. J. Nurs..

[57] Badawy J., Nguyen O.K., Clark C., Halm E.A., Makam A.N. (2017). Is everyone really breathing 20 times a minute? Assessing epidemiology and variation in recorded respiratory rate in hospitalised adults. BMJ Qual. Saf..

[58] Ludikhuize J., Smorenburg S.M., de Rooij S.E., de Jonge E. (2012). Identification of deteriorating patients on general wards; measurement of vital parameters and potential effectiveness of the Modified Early Warning Score. J. Crit. Care.

[59] Palmer J.H., James S., Wadsworth D., Gordon C.J., Craft J. (2023). How registered nurses are measuring respiratory rates in adult acute care health settings: An integrative review. J. Clin. Nurs..

[60] Harry M.L., Heger A.M.C., Woehrle T.A., Kitch L.A. (2020). Understanding Respiratory Rate Assessment by Emergency Nurses: A Health Care Improvement Project. J. Emerg. Nurs..

[61] Ansell H., Meyer A., Thompson S. (2014). Why don′t nurses consistently take patient respiratory rates?. Br. J. Nurs..

[62] Flenady T., Dwyer T., Applegarth J. (2017). Accurate respiratory rates count: So should you!. Australas. Emerg. Nurs. J..

[63] Hogan J. (2006). Why don′t nurses monitor the respiratory rates of patients?. Br. J. Nurs..

[64] Landfeldt E., Edström J., Lindgren P., Lochmüller H. (2017). Patient Preferences for Treatments of Neuromuscular Diseases: A Systematic Literature Review. J. Neuromuscul. Dis..

[65] Santus P., Radovanovic D., Saad M., Zilianti C., Coppola S., Chiumello D.A., Pecchiari M. (2023). Acute dyspnea in the emergency department: A clinical review. Intern. Emerg. Med..

[66] Hashmi M.F., Modi P., Basit H., Sharma S. (2024). Dyspnea. StatPearls.

[67] Ahmed A., Irving S.J. (2023). Approach to the Adult with Dyspnea in the Emergency Department. https://www.uptodate.com/contents/approach-to-the-adult-with-dyspnea-in-the-emergency-department#H1781993866.

[68] Larsson S.C., Burgess S. (2022). Appraising the causal role of smoking in multiple diseases: A systematic review and meta-analysis of Mendelian randomization studies. EBioMedicine.

[69] (2008). A clinical practice guideline for treating tobacco use and dependence: 2008 update. A U.S. Public Health Service report. Am. J. Prev. Med..

[70] Carson K.V., Verbiest M.E., Crone M.R., Brinn M.P., Esterman A.J., Assendelft W.J., Smith B.J. (2012). Training health professionals in smoking cessation. Cochrane Database Syst. Rev..

[71] Stead L.F., Buitrago D., Preciado N., Sanchez G., Hartmann-Boyce J., Lancaster T. (2013). Physician advice for smoking cessation. Cochrane Database Syst. Rev..

[72] Lüthi-Corridori G., Roth A.I., Boesing M., Jaun F., Tarr P.E., Leuppi-Taegtmeyer A.B., Leuppi J.D. (2024). Diagnosis and Therapy of Community-Acquired Pneumonia in the Emergency Department: A Retrospective Observational Study and Medical Audit. J. Clin. Med..

[73] Schnyder D., Lüthi-Corridori G., Leuppi-Taegtmeyer A.B., Boesing M., Geigy N., Leuppi J.D. (2023). Audit of Asthma Exacerbation Management in a Swiss General Hospital. Respiration.

[74] Reyes F.M., Modi P., Le J.K. (2024). Lung Exam. StatPearls.

[75] Shellenberger R.A., Balakrishnan B., Avula S., Ebel A., Shaik S. (2017). Diagnostic value of the physical examination in patients with dyspnea. Clevel. Clin. J. Med..

[76] Clyne B., Olshaker J.S. (1999). The C-reactive protein11Clinical Laboratory in Emergency Medicine is coordinated by Jonathan S. Olshaker, MD, of the University of Maryland Medical Center, Baltimore, Maryland. J. Emerg. Med..

[77] Su Y.-J. (2014). The value of C-reactive protein in emergency medicine. J. Acute Dis..

[78] Ozger H.S., Senol E. (2022). Use of infection biomarkers in the emergency department. Turk. J. Emerg. Med..

[79] Rafie N., Kashou A.H., Noseworthy P.A. (2021). ECG Interpretation: Clinical Relevance, Challenges, and Advances. Hearts.

[80] Riishede M., Lassen A.T., Baatrup G., Pietersen P.I., Jacobsen N., Jeschke K.N., Laursen C.B. (2021). Point-of-care ultrasound of the heart and lungs in patients with respiratory failure: A pragmatic randomized controlled multicenter trial. Scand. J. Trauma. Resusc. Emerg. Med..

[81] Zanobetti M., Poggioni C., Pini R. (2011). Can chest ultrasonography replace standard chest radiography for evaluation of acute dyspnea in the ED?. Chest.

[82] Zanobetti M., Scorpiniti M., Gigli C., Nazerian P., Vanni S., Innocenti F., Stefanone V.T., Savinelli C., Coppa A., Bigiarini S. (2017). Point-of-Care Ultrasonography for Evaluation of Acute Dyspnea in the ED. Chest.

[83] Moore C.L., Copel J.A. (2011). Point-of-care ultrasonography. N. Engl. J. Med..

[84] Smallwood N., Matsa R., Lawrenson P., Messenger J., Walden A. (2015). A UK wide survey on attitudes to point of care ultrasound training amongst clinicians working on the Acute Medical Unit. Acute Med..

[85] Lichtenstein D.A., Mezière G.A. (2008). Relevance of lung ultrasound in the diagnosis of acute respiratory failure: The BLUE protocol. Chest.

[86] Brenner D.J., Hall E.J. (2007). Computed Tomography—An Increasing Source of Radiation Exposure. N. Engl. J. Med..

